# Increased Intraplatelet ADMA Level May Promote Platelet Activation in Diabetes Mellitus

**DOI:** 10.1155/2020/6938629

**Published:** 2020-09-29

**Authors:** Jakub Gawryś, Jerzy Wiśniewski, Ewa Szahidewicz-Krupska, Damian Gajecki, Julia Leśniewska, Filip Majda, Karolina Gawryś, Paulina Fortuna, Piotr Mlynarz, Adrian Doroszko

**Affiliations:** ^1^Department of Internal Medicine, Hypertension and Clinical Oncology, Faculty of Medicine, Wroclaw Medical University, Wroclaw, Poland; ^2^Department of Medical Biochemistry Faculty of Medicine, Wroclaw Medical University, Wroclaw, Poland; ^3^Department of Biochemistry, Molecular Biology and Biotechnology, Faculty of Chemistry, Wroclaw University of Science and Technology, Wroclaw, Poland

## Abstract

**Background:**

Antiplatelet therapy has become a standard therapeutic approach in the secondary prevention of cardiovascular system disorders of thrombotic origin. Patients with concomitant diabetes mellitus (DM) obtain fewer benefits from this treatment. Hence, the pathophysiology of altered platelet function in response to glucose metabolism impairment should be of particular interest.

**Objectives:**

The aim of our study was to verify if the platelet expression of the asymmetric dimethylarginine (ADMA) in diabetic patients differs in comparison to the nondiabetic ones. The correlation of platelet-ADMA with platelet activation and aggregation as well as with other risk factors was also investigated. *Material and Methods*. A total of 61 subjects were enrolled in this study, including thirty-one type 2 diabetic subjects without diabetes-related organ damage. Physical examination was followed by blood collection with an assessment of platelet aggregation, traditional biochemical cardiovascular risk factors, and evaluation of nitric oxide bioavailability parameters in plasma and thrombocytes. Subsequently, the assessment of endothelial function using Peripheral Arterial Tonometry and Laser Doppler Flowmetry (LDF) was performed.

**Results:**

In the DM group, elevated concentration of intraplatelet ADMA and higher ADMA/SDMA ratio compared to the control group was observed. It was accompanied by higher ADP-mediated platelet aggregation and lower microvascular response to a local thermal stimulus measured by LDF in the diabetes group.

**Conclusions:**

Type 2 diabetes is related to higher intraplatelet concentration of asymmetric dimethylarginine (ADMA), which may result in impaired platelet-derived nitric oxide synthesis and subsequent increased platelet activity, as assessed by the ADP-induced aggregation. Laser Doppler Flowmetry, compared to EndoPAT 2000, appears to be a more sensitive indicator of the impaired microvasculature vasodilation in diabetics without the presence of clinically significant target organ damage.

## 1. Introduction

**Figure 1:**
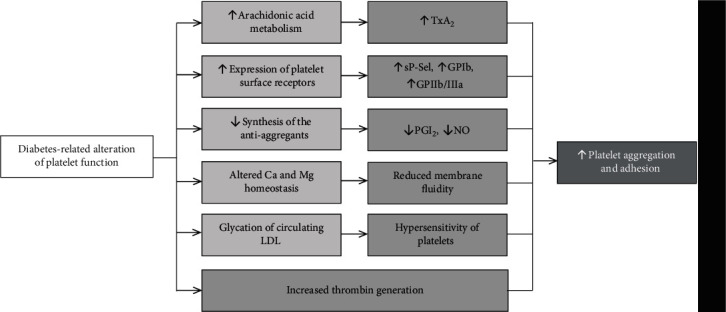
Summary of diabetes-related alterations of platelet function. TXA2: thromboxane A2; sP-Sel: P-selectin soluble form; GPIb: platelet glycoprotein Ib; GPIIb/IIIa: platelet glycoprotein IIb/IIIa; PGI2: prostacyclin; NO: nitric oxide; LDL: low density lipoprotein. Modification based on 12^,^^13^

Antiplatelet therapy, after several decades following its implementation to clinical practice, has become a standard therapeutic approach in preventing cardiovascular events of thrombotic origin 1. Based on numerous clinical studies, it has been clearly demonstrated that it markedly lowers the morbidity and mortality in the high-risk population 2. Nevertheless, patients with concomitant diabetes mellitus, being at least at high cardiovascular risk, obtain fewer benefits from this treatment, when compared to the nondiabetics. This partially explains the higher incidence of cardiovascular events in this subpopulation 3. Among numerous factors (Figure 1), increased platelet activity, platelet aggregation, and altered platelet-endothelial cell interaction may play a key role in developing vascular diabetic complications 4, 5. Hence, the pathophysiology of altered platelet function in response to the presence of glucose metabolism abnormalities should be of particular interest in developing novel therapeutic approaches minimizing the occurrence of acute cardiovascular events in the diabetic population.

Nitric oxide (NO) and the regulation of its synthesis play a crucial role in the pathogenesis of cardiovascular disorders. Over the last three decades, numerous studies described the exact role of NO and its metabolic pathway components in developing endothelial dysfunction and its contribution to further cardiovascular events of ischemic origin^6^. A competitive nitric oxide synthase inhibitor, asymmetric dimethylarginine (ADMA), appears to be a key element regulating the NO bioavailability, and its elevated plasma level was shown in numerous studies to be an early predictor for increased cardiovascular risk and future cardiovascular events 7–9. ADMA was demonstrated to be present not only in plasma but also in the intracellular compartment, including endothelial cells and human platelets 10.

Platelet-derived nitric oxide (PDNO) reduces the platelet degranulation, decreases thrombocyte and leukocyte adhesion to endothelium, and finally limits the aggregation process 11. Differences in regulation and alterations in its metabolism may result in increased platelet aggregation and contribute to their resistance to the antiplatelet treatment, observed among the populations at high risk for cardiovascular events 12, 13. As the role of PDNO appears to be partially different from the endothelial-derived NO (primarily associated with vasodilation), it might be considered as the autonomous system involved in the maintenance of intravascular homeostasis 11. The determination of the intraplatelet parameters of the NO metabolic pathway and their relation to the same parameters measured in plasma may contribute to a better understanding of pathophysiological processes associated with increased cardiovascular risk in diabetes.

The population of patients with diabetes mellitus is continuously growing. Recently, the heterogeneity of this group and the need for an individual approach have been increasingly emphasized. It is expected that in almost 30% of cases a 5-year CVD risk in diabetic patients is similar to the general population 14. Therefore, a more precise estimation of the long-term risk for cardiovascular events is required. In this purpose, the methods for the assessment of endothelial function, which are proven to have an additional predictive value comparing to traditional risk factors^15^, such as EndoPAT 2000 and Laser Doppler Flowmetry, are used. Although both methods examine the endothelial function by evaluating changes in peripheral microcirculation, recent reports indicate their dependence on different mechanisms regulating its vasodilatory function 16.

Hence, the aim of our study was to verify if the platelet expression of the competitive nitric oxide synthase inhibitor, ADMA in diabetic patients, differs in comparison to the nondiabetic ones. The relations between intraplatelet ADMA levels and platelet activation properties were verified. The possible cross-talk between platelet ADMA and the occurrence of other cardiovascular risk factors as well as the presence of endothelial vasodilatory dysfunction were other goals investigated in this study.

## 2. Material and Methods

### 2.1. Bioethics Approval

The study protocol and all experiments were investigated and approved by the local Bioethics Committee. All of the volunteers agreed to participate in the study by signing a written informed consent, which was also verified by the Bioethical Committee. The project procedures are consistent with the principles of the Declaration of Helsinki (Seventh Revision, 64^th^ World Medical Association meeting, Fortaleza, 2013).

### 2.2. Subject Recruitment and Examination

**Figure 2:**
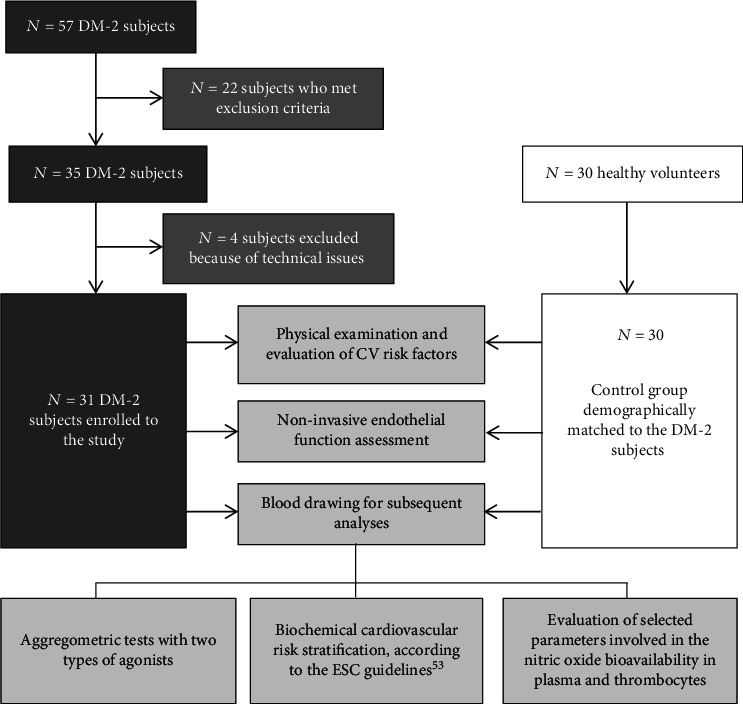
Study protocol. DM-2: type 2 diabetes mellitus; CVD: cardiovascular diseases; NOS: nitric oxide synthase.

87 subjects were considered to participate in this study. After careful analysis of the inclusion and exclusion criteria followed, a total of 61 subjects were enrolled in this study, including thirty-one type 2 diabetic subjects aged 35-70, in the phase of oral treatment and without significant diabetes-related target organ damage. The control group constituted 30 healthy volunteers demographically matched to the study group in whom the glucose metabolism alterations, including diabetes, impaired fasting glycemia, impaired glucose tolerance, and insulin resistance, were excluded. All of the study participants underwent a standard detailed physical examination. Afterwards, 44 ml of peripheral venous blood was collected atraumatically by the Sarstedt S-Monovette® (Sarstedt Ag & Co., Nümbrecht, Germany) aspiration and vacuum kit for subsequent biochemical analyses. In both groups, these procedures were followed by biometric measurements and assessment of endothelial vasodilatory function using the Laser-Doppler Flowmetry (PeriFlux 5000, Perimed AB, Järfälla, Sweden) and Peripheral Artery Tonometry (EndoPAT 2000, Itamar Medical, Caesarea, Israel). From collected blood, biochemical characterization of both groups, including the determination of basic cardiovascular risk factors, aggregometric tests, and evaluation of selected parameters involved in the nitric oxide biotransformation pathway, was performed. The study protocol is summarized in Figure 2.

### 2.3. Platelet Preparation for LC-MS Analysis

The collected whole blood was supplemented with prostacyclin (PGI_2_) at the final concentration of 0.06 *μ*g/ml and centrifuged for 20 minutes at 230 × g at 21°C to obtain platelet-rich plasma. Subsequently, PRP was supplemented with PGI_2_ (final concentration 0.3 *μ*g/ml) and centrifuged for 10 minutes at 1000 × g at 21°C. The plasma was discarded, and the platelet pellet was carefully washed three times with 1 ml of Tyrode HEPES buffer pH 7.4. Rinsed platelets were suspended in 4 ml of Tyrode HEPES buffer pH 7.4 supplemented with CaCl_2_ (final concentration 1 mM). The resulted suspension was immediately analyzed for platelet count and contamination with WBC and RBC (Sysmex device, Sysmex, Norderstedt, Germany). The pure PLT suspension was adjusted with Tyrode HEPES buffer pH 7.4 containing CaCl_2_ to a final concentration of 2.5 × 10^8^ ml. Samples containing platelets in amounts of 5.0 × 10^8^ cells were preserved for LC-MS analysis. The samples were obtained by centrifugation of the suspension of known concentration for 5 min, 10000 × g at 4°C, and stored at −80°C until further analyses.

### 2.4. Evaluation of Intraplatelet Parameters of the Nitric Oxide Bioavailability

Into platelet pellet, 10 *μ*l of internal standard solution and 700 *μ*l of cold extraction solution consisting of methanol and water (7 : 3) were added. Samples were then vortexed (5 min, 2000 rpm, 4°C), frozen (-20°C, 15 min), and centrifuged (9 min, 15000 rcf, 4°C). Subsequently, supernatants were transferred to the new polypropylene microtubes and dried at 55°C using SpeedVac evaporator (Thermo Savant). Dried samples were then derivatized as bellow.

LC-MS/MS analysis was performed using the Acquity UPLC system (Waters, Milford, MA, USA) equipped with a cooled autosampler. The sample temperature in the autosampler was set to 6°C, and the injection volume was 4 *μ*l. The Waters BEH Shield C18 column (1.7 *μ*m, 2.1 × 10 mm) was thermostatted in a column oven at 60°C. The flow rate was 0.350 ml/min with a total run time of 8 min. Eluents: A: water with 0.1% formic acid (FA), B: methanol with 0.1% FA. The following gradient was used: 0.0 min–3% B, 2.5 min–14% B, 4.6 min–60% B, 4.8 min–90% B, 6.1 min–3% B. MS analysis was performed using SYNAPT G2 Si mass spectrometer (Waters, Milford, MA, USA) equipped with electrospray ionization source (ESI) in positive ionization mode. The spray voltage, source temperature, and the desolvation temperature were set at 0.5 kV, 140°C, and 450°C, respectively. Data acquisition was performed using MassLynx software (Waters) for the following ions (m/z): 150.0919, 156.1295, 237.1239, 243.1339, 263.1090, 267.1382, 279.1457, 286.1897, 307.1770, and 314.2209 for DMA, D6-DMA, ornithine, D6-ornithine, citrulline, D4-citrulline, arginine, D7-arginine, ADMA, SDMA, and D7-ADMA, respectively. Standard calibration curves were prepared using the following concentration ranges: 0.3 to 15 *μ*M for ornithine, 0.5 to 25 *μ*M for arginine, 0.005 to 0.25 *μ*M for ADMA and SDMA, 0.1 to 5 *μ*M for citrulline, and 0.014 to 0.7 *μ*M for DMA.

### 2.5. Evaluation of Parameters of the Nitric Oxide Bioavailability in Plasma

The plasmatic level of metabolites involved in NO synthesis was measured according to the method described by Fleszar et al. 17. Briefly, 100 *μ*L of plasma, 50 *μ*l of borate buffer, and 10 *μ*l of internal standard solution (100 *μ*M D7-arginine, 20 *μ*M D7-ADMA, 25 *μ*M D6-DMA, 100 *μ*M D6-ornithine, and 50 *μ*M D4-citrulline) were transferred into 2 ml polypropylene tubes and mixed (1 min, 1200 RPM, 25°C). Then, 400 *μ*l of acetonitrile and 10 *μ*l of 10% BCl in acetonitrile were added and mixed (10 min, 1200 RPM, 25°C). Subsequently, samples were centrifuged (7 min, 4°C, 22,000 RCF) and 100 *μ*l of clear supernatant was diluted four times with water, transferred to chromatographic glass vials, and analyzed. LC-MS/MS analysis was performed using the equipment and settings described above with 10-fold higher concentrations ranges of standard calibration curves.

### 2.6. Aggregometry

Platelet function was assessed using the impedance aggregation method in whole blood using the four-channel optical aggregometer (Chrono-log 700, Chrono-Log, Pennsylvania, USA). This method is based on multiple platelet aggregation on the electrodes and changing of the electrical resistance between their two wires. Whole blood was collected to the polypropylene tubes for 10% sodium citrate using the Sarstedt S-Monovette® (Sarstedt Ag & Co., Nümbrecht, Germany) aspiration and vacuum kit. After collection, the tubes were kept at room temperature for a maximum of 90 minutes before engaging the test. Two different aggregation activators were used: adenosine diphosphate (ADP) and arachidonic acid (AA). The 1 : 1 solution of whole blood in room temperature with 0,9% natrium chloride was placed in the test chambers. Then, a certain number of agonists, necessary to obtain appropriate concentrations (0,5 mg/ml for AA, 20 *μ*mol for ADP), was added to the prepared solution. After 6 minutes, aggregation curves were recorded, measured, and analyzed by dedicated software (Aggrolink®, Chrono-Log, Pennsylvania, USA). The increase in electrical impedance was given in aggregation units.

### 2.7. Endothelial Function Assessment

**Figure 3:**
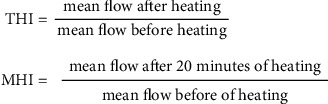
Calculation of microvascular flow parameters changes measured by Laser Doppler Flowmetry. THI: total hyperemia index; MHI: maximum hyperemia index.

The ability of the endothelium to dilate blood vessels through the synthesis of nitric oxide was measured by two devices following two different protocols. Laser Doppler Flowmetry allows for dynamic monitoring of changes in the microcirculation of superficial skin layers under the effect of stimuli: chemical (like acetylcholine or pilocarpine) or thermal—through a heated thermostatic probe. The wavelength of the laser beam changes when it hits in the moving blood cells and the changes in its frequency and distribution are associated with the number and speed of blood morphotic elements flowing through the vessels. In this study, the local thermal stimulus was used. Standard recording during local heating consists of two main phases: peak phase (up to several minutes)—dependent on stimulation of local sensory nerves followed by substance P excretion, and plateau phase (after 20 minutes)—conditioned mostly by nitric oxide and partially by norepinephrine and neuropeptide Y ^18^. The probe of the LDF device (Periflux 5000, Perimed, Järfälla, Sweden) was placed on the forearm skin with no visible superficial vessels. The studied limb was immobilized with a vacuum pillow provided by the device manufacturer. After 10 minutes of baseline record with a temperature of 33°C, heating was set to 44°C for the next 30 minutes. To prevent the impact of baseline flow variability, the results were shown as total hyperemia index (THI) and maximum hyperemia index (MHI) (Figure 3).

Simultaneously, the endothelial response to transient hypoxemia was measured by Peripheral Arterial Tonometry (EndoPAT 2000, Itamar Medical, Caesarea, Israel). Microvascular blood flow in both hands' middle fingers was measured by dedicated pressure probes. These sensors are equipped with a latex cuff which, when inflated, through the pressure change generated by the blood flow, generates a signal that is converted by dedicated computer software into a pulse waveform. This signal was recorded for 6 minutes before and after five minutes of ischemia induced by inflation of a sphygmomanometer cuff minimum 50 mmHg over the systolic blood pressure value as described in the protocol developed by the device manufacturer. At least 15 min prior and during the examination patient remained in the isolated quiet room, with constant air temperature, and was instructed to move as little as possible to prevent signal disturbances. As a result, the software created by Itamar calculates the reactive hyperemia index (RHI) and augmentation index (AI).

### 2.8. Statistical Analysis

The data is presented as the mean ± SD. The differences between two continuous parameters were assessed using a Mann-Whitney *U*-test or a Student's *t*-test, following the Shapiro-Wilk test and Levene's test as appropriate. For comparison of more than two groups, an ANOVA followed by Tukey's test or a Friedman test (for nonparametric statistics) was performed as appropriate. All calculations were made with Statsoft® Statistica 13.3 software.

## 3. Results

### 3.1. Baseline Characteristics

The characteristics of the diabetic and control groups are presented in Table 1. The groups were matched in both age and sex. Regarding demographical measurements, higher BMI and WHR ratios were observed in the DM group. In morphological parameters, only WBC was significantly higher in the DM group, but its value did not exceed the normal range. The difference in HBA_1C_, fasting glucose, and insulin level with higher rates in DM subjects was also observed. According to biochemical characteristics, differences involved only eGFR, hsCRP, serum sodium, and magnesium, but the mean values did not extend the reference limits. Higher HOMA-IR and lower QUICKI indexes were noted is the DM group.

### 3.2. Assessment of Endothelial Function

There were no significant differences between groups in the Reactive Hyperaemia Index (RHI) and Augmentation Index (AI), as measured by the EndoPAT® 2000. However, the change in superficial blood flow following the local thermal stimulus revealed significantly lower values of both calculated hyperemia indexes in diabetic, as examined by Laser Doppler Flowmetry. A summary of the endothelial function assessment is shown in Table 2.

Abbreviations: RHI: reactive hyperemia index (EndoPAT 2000); AI: augmentation index (EndoPAT 2000); THI: total hyperemia index (Laser Doppler Flowmetry); MHI: maximum hyperemia index (Laser Doppler Flowmetry).

### 3.3. Parameters of the Platelet and Plasma NO Bioavailability

**Figure 4:**
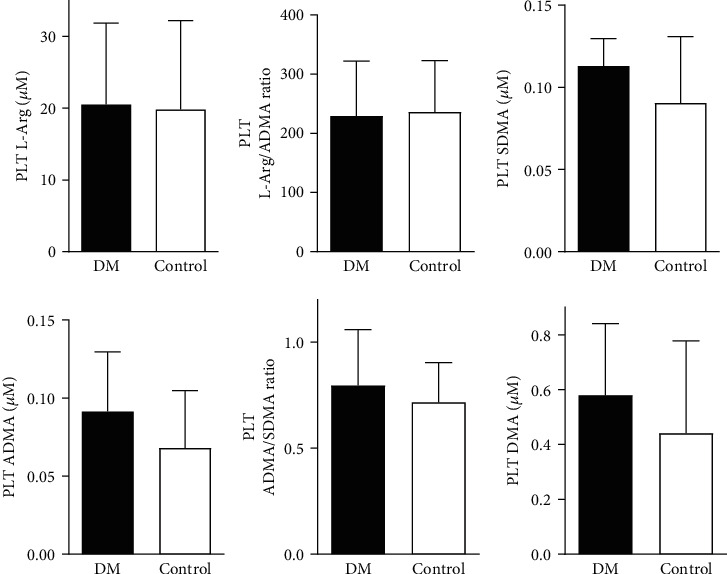
The comparison of intraplatelet parameters of nitric oxide bioavailability. ^∗^*p* < 0, 05 versus control group. Abbreviations: PLT L-Arg: intraplatelet L-arginine; PLT ADMA: intraplatelet asymmetric dimethylarginine; PLT SDMA: intraplatelet symmetric dimethylarginine; PLT DMA: intraplatelet dimethylamine; DM: diabetes mellitus group; Control: control group; PLT ADMA: intraplatelet asymmetric dimethylarginine.

**Figure 5:**
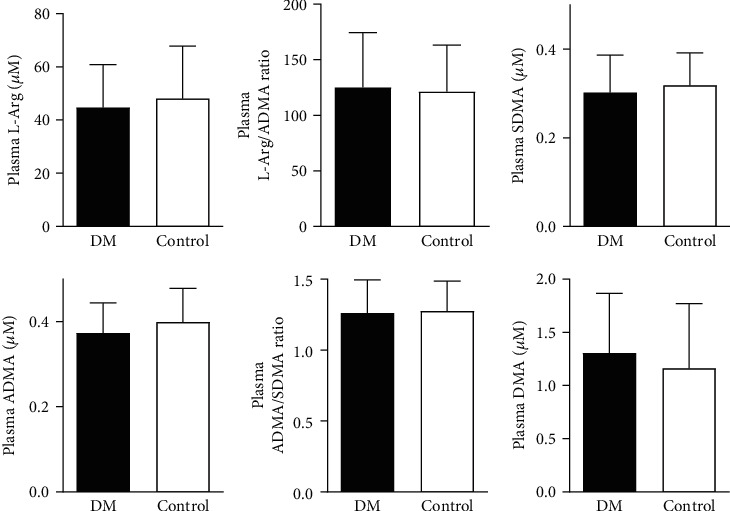
The comparison of plasmatic parameters of nitric oxide bioavailability. Abbreviations: Plasma L-Arg: plasmatic L-arginine; Plasma ADMA: plasmatic asymmetric dimethylarginine; Plasma SDMA: plasmatic symmetric dimethylarginine; Plasma DMA: plasmatic dimethylamine; DM: diabetes mellitus group; Control: control group;

In the DM group, elevated concentrations of intraplatelet ADMA (0,091 ± 0,038 *μ*M versus 0,068 ± 0,037 *μ*M, *p* = 0,003), DMA (0, 58 ± 0, 26 versus 0, 44 ± 0, 33 *μ*M, *p* = 0,049), and higher ADMA/SDMA ratio (0, 80 ± 0, 26 versus 0, 72 ± 0, 19, *p* = 0,046) compared to the control group were shown (Figure 4.). There were no differences in other studied intraplatelet parameters of the nitric oxide bioavailability (L-Arg, SDMA, and L-Arg/ADMA) (Figure 4) and in all investigated plasma parameters (Figure 5.).

### 3.4. Aggregometry

**Figure 6:**
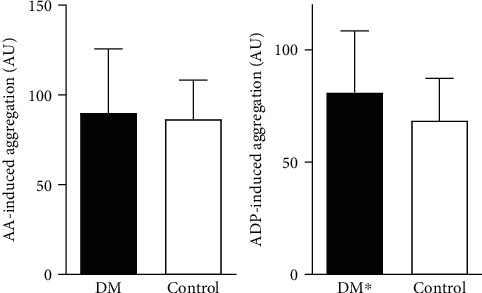
The comparison of platelet aggregation induced by AA and ADP. ^∗^*p* < 0, 05 versus control group. Abbreviations: AA: arachidonic acid; ADP: adenosine diphosphate; DM: diabetes mellitus group; Control: control group.

Significantly higher aggregation in response to ADP was observed in diabetic subjects when compared to the controls (83, 58 ± 23, 68 versus 68, 43 ± 18, 97 U, *p* = 0,017) (Figure 6.). There was no difference in aggregation stimulated by arachidonic acid between studied groups.

In the diabetic group, a positive correlation between platelet ADMA and serum calcium (*r* = 0, 41), potassium (*r* = 0, 44), as well as platelet SDMA concentration (*r* = 0, 67) and age (*r* = 0, 41) were noted. What is more, a moderate negative correlation with serum magnesium (*r* = –0, 55) was demonstrated. The positive correlation was present between ADP stimulated aggregation with serum calcium level (*r* = 0, 41) and platelet SDMA concentration (*r* = 0, 43). A negative correlation was shown between diabetes-related parameters (glycemia, HbA_1C_) and platelet SDMA concentration. In the control group, platelet ADMA concentration significantly correlated with platelet SDMA (*r* = 0, 88), DMA (*r* = 0, 69), and plasma ADMA (0,55).

## 4. Discussion

This is the first study to assess the parameters of the L-arginine: nitric oxide metabolic pathway in diabetic human platelets in comparison to the non-diabetic control. The enrolled groups were homogenous in all, except for diabetes-related biochemical parameters (glucose and insulin concentration, HbA_1C_). Discrepancies in other parameters (hsCRP, eGFR, and sodium) did not extend the normal values.

Numerous studies link elevated plasma ADMA concentration with both type 1 and 2 diabetes. As it is reported, a higher ADMA level in this group of patients is related to the development of retinopathy 19, progression of nephropathy 20, and may be a predictor of future cardiovascular events 21. However, our results show no significant difference in the plasma concentration of ADMA and other measured nitric oxide metabolic pathway components in diabetic subjects, compared to the control group. According to Xiong et al., plasma ADMA concentrations are not dependent on the duration of the disease but are related to the existence of macroangiopathy 22. Other reports underline the predictive value of increased plasmatic ADMA and hsCRP concentrations in cardiovascular events in diabetes, however, the values of these parameters were higher than in our study (>0,63 *μ*mol/l for ADMA, >6,0 mg/l for hsCRP) 21. Thus, the lack of difference observed in our study can be described by the early stage of the disease and the absence of diabetes-related organ damage.

In contrary to plasmatic ADMA, we demonstrated that intraplatelet ADMA concentration was significantly higher in patients with type 2 diabetes. This result was accompanied by a significantly higher concentration of dimethylamine (DMA)—a product of ADMA degradation catalyzed by the dimethylaminohydrolase (DDAH). As ADMA is the most potent inhibitor of all isoforms of nitric oxide synthase 23, which lower intraplatelet activity was recognized in previous studies 24, 25, there is a high probability that this mechanism is responsible for platelet-derived NO deficiency in diabetic subjects. What is more, PDNO-derived activation of intraplatelet cyclic guanidine monophosphate (cGMP) can cause platelet degranulation and aggregation 26, instead of inhibiting thrombocytes' activation. This statement is supported by the increased aggregation observed in the c-GMP-dependent phosphokinase A- (PKA-) knockout mice 26. Inhibition of platelet degranulation occurs with higher intraplatelet concentrations of NO by nitrosylation of soluble N-ethylmaleimide-sensitive factor (NSF) which prevents from the attachment to its receptors (SNAREs) involved in the process of platelet granules exocytosis 27. Considering that, it could be another mechanism affecting platelet function dependent on intraplatelet NO deficiency. Despite the fact that higher concentrations of L-arginine enhance nitric oxide synthesis 28 by increasing the substrate availability and the L-arginine/ADMA ratio, our results demonstrated no significant difference in intraplatelet concentrations of these parameters between studied groups. It indicates that the substrate/inhibitor relationship is not the sole determinant of its bioavailability without considering the membrane migration and degradation processes. Given the complexity of the intraplatelet nitric oxide synthesis pathway, more detailed research including the determination of all components involved in intraplatelet L-arginine transport, NOS activity regulation, and NO/cGMP pathway is necessary.

Interestingly, we observed no correlation between intraplatelet and plasma ADMA concentrations. The growing data suggests that these two compartments are independent of each other 11. Hence, it appears that the intraplatelet ADMA accumulation, potentially decreasing platelet-derived nitric oxide production, occurs at the earlier stages of diabetes than in plasma. As intraplatelet ADMA concentration correlates with the concentration of serum magnesium and calcium, the transport alterations might be involved in this process, but these mechanisms have yet to be explored.

As results from our study, a significant difference in aggregation mediated by ADP between groups with higher values in the diabetic one was observed, which is in line with the work of other authors 29, 30. What is more, some studies indicate that almost two-thirds of diabetic subjects were resistant to antiplatelet therapy with ADP-antagonist—clopidogrel 31, 32. The impairment of the PDNO production is postulated to be one of the main reasons for platelet hyperaggregability 11, thus elevated intraplatelet ADMA concentration, by competitive eNOS inhibition, may be considered as a primary cause of this condition. A potential cross-talk between these two processes may occur in the level of cGMP and cAMP interaction. Fleming et al. investigated the effect of insulin on activation of intraplatelet eNOS and the production of nitric oxide and demonstrated it to stimulate both cyclic GMP and AMP formation 33, which the action inhibits thrombocyte aggregation. Stimulating the P2Y_12_ receptor with ADP, *via* the Gi protein action, lowers the concentration of intraplatelet cAMP leading to platelet aggregation 34. Therefore, lower PDNO concentration due to intraplatelet ADMA accumulation could be the reason for lower basal cAMP concentration, which results in increased aggregation with ADP whose activity is conducted by interaction with the same intracellular signal transmitter. On the contrary, despite the theoretical consistency, we found no significant correlation between intraplatelet ADMA concentration and aggregation induced by both ADP and arachidonic acid. However, a close relationship between the tested parameters cannot be excluded, possible involvement of other mechanisms of increased platelet aggregability in diabetes, for example, reduced platelet membrane lipid fluidity 35 or the action of advanced glycation end products (AGEs) 36, should be considered.

Noteworthy, we found a moderate negative correlation between serum magnesium and intraplatelet ADMA. The difference in serum magnesium concentration among studied groups may be the result of the increased Mg^2+^ transport from extracellular to the intracellular compartment in insulin-enhanced mechanism 37, 38. What is more, hypomagnesemia has been connected with impaired vasodilation and decrease in NO release 39, which would be consistent with the results of the current work, where lower serum magnesium levels are correlated with higher intraplatelet ADMA concentrations inhibiting the nitric oxide synthase. However, some authors showed that Mg^2+^-induced vasodilation is independent of NO^40^, and others suggest that low magnesium prevents only from nitric oxide release, not production^39^, so its precise role in this pathway remains unclear.

In the current study, diabetic subjects were also characterized by elevated PLT ADMA/SDMA ratio in comparison with the control group. These results are accompanied by the difference between intraplatelet ADMA and SDMA in both studied groups. In both groups, these parameters significantly correlated with each other. In the control group, the correlation is very strong when in diabetic subjects, it decreases to moderate. What is more, the intraplatelet SDMA concentration negatively correlates with both glucose and HBA_1C_ level. It allows for careful speculation, that hyperglycemia, by inducing the protein glycation, may change the proteins' affinity to methyltransferases (PMRT-I, PMRT-II) and alter the process of their arginine residues methylation. The possible influence of platelet protein glycation on thrombocyte function has been repeatedly highlighted in scientific reports 25, 41, and its potential impact on NO production was also considered 42. Besides, glycation occurs between carbonyl groups and free amino groups present in polypeptide chain 43. Since arginine belongs to basic amino acids, its free amino group is a potential site of nonenzymatic glucose and another monosaccharide attachment. In the same location, the methyl group is attached by the action of PMRT 44. Therefore, the balance of the proteins' methylation may be disturbed to the side of asymmetrical methylation at the expense of symmetrical one, which results in the formation of more asymmetrical arginine derivatives after proteolysis causing the increase of ADMA/SDMA ratio and intraplatelet ADMA concentration, which inhibits NOS activity and thus reduces PDNO production. This theory is supported by the study of Lee et al. that arginine residue methylation is altered during the development of diabetes 45. Changes in arginine methylation are not only related to the formation of methylated arginine-derivates but also are known as an epigenetic regulator of gene transcription, cell signalling, protein stability, and translation 46, 47

In the current study, we assessed endothelial vasodilatory function using EndoPAT 2000 and Laser Doppler Flowmetry (LDF). According to Jakubowski et al., these methods evaluate independent mechanisms regulating the profile of endothelial vasodilatory function 16. We have shown reduced microvascular response to local peripheral thermal stimulus measured by LDF in diabetic subjects in comparison to the control group with no significant difference in the indexes provided by EndoPAT 2000. These results did not correlate with any of the demographical or biochemical risk factors what is in line with the findings of previously cited author. As vasodilation caused by the thermal stimulus applied locally is dependent not only on the activity of nitric oxide produced by eNOS isoform 48 but also on sensory nerve stimulation and release of sympathetic nervous system neurotransmitters 18. Both described mechanisms might be involved in impaired response to thermal stimulus in diabetic patients 49, 50. In diabetes, changes in microvascular flow measured by LDF, as shown in current and other studies 49, 51, were detected in earlier stages of the disease compared to EndoPAT 2000 52, which allows considering Laser Doppler Flowmetry as a more sensitive indicator of microvascular status. However, the exact role of the LDF as an independent predictor of cardiovascular events requires further investigation.

To conclude, type 2 diabetes is related to higher intraplatelet concentration of asymmetric dimethylarginine (ADMA), which may result in impaired platelet-derived nitric oxide synthesis and subsequent increased platelet activity, as assessed by the ADP-induced aggregation. In the early stage of well-controlled diabetes type 2, the accumulation of intraplatelet ADMA occurs prior to its increase in plasma. What is more, the Laser Doppler Flowmetry, but not EndoPAT 2000, showed decreased microvascular skin flow after local heating in the diabetic group, hence LDF appears to be a more sensitive indicator of the impaired microvasculature vasodilation in diabetics without the presence of clinically significant target organ damage.

## Figures and Tables

**Table 1:** Demographic and biochemical characteristics between studied groups including cardiovascular risk stratification parameters. Results are presented as mean ± SD.

Parameter	Diabetes group *N* = 31 (mean ± SD)	Control group *N* = 30 (mean ± SD)	*p* value
Age (y)	58, 48 ± 8, 17	54, 67 ± 6, 08	NS
Women (%)	10 (32%)	13 (43%)	NS
BMI (kg/m^2^)	30, 18 ± 3, 94	26, 02 ± 3, 87	<0,05
WHR	0, 98 ± 0, 08	0, 92 ± 0, 12	<0,05
WBC (k/*μ*l)	7, 18 ± 2, 13	5, 89 ± 1, 57	<0,05
RBC (mln/*μ*l)	4, 72 ± 0, 48	4, 88 ± 0, 50	NS
Haemoglobin (g/dl)	14, 35 ± 1, 25	14, 68 ± 1, 26	NS
Haematocrit (%)	42, 15 ± 3, 62	43, 70 ± 3, 75	NS
MCV (fL)	89, 4 ± 4, 64	89, 69 ± 3, 94	NS
MCH (pg)	30, 47 ± 2, 00	30, 16 ± 1, 32	NS
MCHC (g/dL)	34, 07 ± 1, 08	33, 63 ± 0, 98	NS
Platelets (k/*μ*l)	250,81 ± 57, 36	244 ± 40, 59	NS
PDW (fL)	13, 07 ± 2, 63	12, 51 ± 1, 85	NS
Glucose (mg/dl)	115,73 ± 40, 91	93, 82 ± 8, 79	<0,05
Hb1_AC_ (%)	6, 13 ± 0, 60	5, 56 ± 0, 30	<0,05
Insulin (uU/mL)	8, 79 ± 4, 93	6, 71 ± 3, 60	<0,05
Total cholesterol (mg/dl)	194,06 ± 52, 17	220,76 ± 49, 55	NS
LDL (mg/dl)	114,29 ± 44, 03	136,31 ± 39, 25	NS
HDL (mg/dl)	50, 13 ± 12, 05	57, 24 ± 14, 50	NS
Triglycerides (mg/dl)	164,10 ± 104,45	135,93 ± 68, 79	NS
hsCRP (mg/l)	2, 46 ± 2, 53	1, 05 ± 0, 82	<0,05
Creatinine (mg/dl)	0, 91 ± 0, 15	0, 96 ± 0, 16	NS
eGFR (ml/min/1,73m^2^)	84, 57 ± 14, 52	77, 86 ± 10, 24	<0,05
Urea (mg/dl)	33, 97 ± 6, 83	34, 20 ± 8, 74	NS
Uric acid (mg/dl)	6, 05 ± 1, 50	5, 54 ± 1, 44	NS
TSH (*μ*IU/mL)	1, 79 ± 0, 92	1, 60 ± 0, 56	NS
Troponin I (pg/ml)	3, 08 ± 2, 06	2, 32 ± 0, 98	NS
BNP (pg/ml)	28, 98 ± 36, 89	21, 36 ± 15, 36	NS
Sodium (mmol/l)	139,33 ± 1, 82	140,55 ± 2, 36	<0,05
Potassium (mmol/l)	4, 33 ± 0, 28	4, 29 ± 0, 31	NS
Magnesium (mg/dl)	1, 94 ± 0, 23	2, 14 ± 0, 16	<0,05
Calcium (mmol/l)	9, 72 ± 0, 38	9, 63 ± 0, 32	NS
HOMA IR	2, 49 ± 1, 31	1, 68 ± 0, 89	<0,05
HOMA-*β* (%)	89, 57 ± 126,66	91, 34 ± 51, 32	NS
QUICKI	0, 60 ± 0, 09	0,668 ± 0, 10	<0,05

Abbreviations: NS: result statistically nonsignificant; BMI: body mass index; WHR: waist-hip ratio; WBC: white blood cells; RBC: red blood cells; MCV: mean (red blood) cell volume; MCH: mean corpuscular haemoglobin; MCHC: mean corpuscular haemoglobin concentration; PDW: platelet distribution width; eGFR: estimated glomerular filtration rate; HDL: high-density lipoprotein; LDL: low-density lipoprotein; hsCRP: high-sensitivity C-reactive protein; TSH: thyroid-stimulating hormone; BNP: brain natriuretic peptide.

**Table 2:** Assessment of endothelial function by EndoPAT 2000 and Laser Doppler Flowmetry in studied groups.

Parameter	Diabetes group	Control group	*p* value
RHI	2, 35 ± 0, 87	2, 23 ± 0, 55	NS
AI (%)	29, 48 ± 19, 84	21, 18 ± 17, 18	NS
THI	7, 77 ± 4, 34	11, 21 ± 6, 87	<0,05
MHI	8, 98 ± 4, 88	14, 63 ± 8, 56	<0,05

## Data Availability

The original data used to support the findings of this study are available from the corresponding author upon request.
